# Continuous Sugarcane Planting Negatively Impacts Soil Microbial Community Structure, Soil Fertility, and Sugarcane Agronomic Parameters

**DOI:** 10.3390/microorganisms9102008

**Published:** 2021-09-23

**Authors:** Ziqin Pang, Muhammad Tayyab, Chuibao Kong, Qiang Liu, Yueming Liu, Chaohua Hu, Jinwen Huang, Peiying Weng, Waqar Islam, Wenxiong Lin, Zhaonian Yuan

**Affiliations:** 1 Key Laboratory of Sugarcane Biology and Genetic Breeding, Ministry of Agriculture, Fujian Agriculture and Forestry University, Fuzhou 350002, China; ziqintea@126.com (Z.P.); tyb.pk@hotmail.com (M.T.); kongchuibao18@163.com (C.K.); fjnldxlq1190101005@163.com (Q.L.); liuyueming12@126.com (Y.L.); chhu@fafu.edu.cn (C.H.); 2 College of Agriculture, Fujian Agriculture and Forestry University, Fuzhou 350002, China; hqliuyh@163.com (J.H.); fzwpy0111@163.com (P.W.); ddoapsial@yahoo.com (W.I.); 3 Fujian Provincial Key Laboratory of Agro-Ecological Processing and Safety Monitoring, College of Life Sciences, Fujian Agriculture and Forestry University, Fuzhou 350002, China; 4 Key Laboratory of Crop Ecology and Molecular Physiology, Fujian Agriculture and Forestry University, Fuzhou 350002, China; 5 Province and Ministry Co-Sponsored Collaborative Innovation Center of Sugar Industry, Guangxi University, Nanning 530000, China

**Keywords:** microbial communities, monoculture practices, soil degradation, sugarcane, high-throughput sequencing

## Abstract

Continuous planting has a negative impact on sugarcane plant growth and reduces global sugarcane crop production, including in China. The response of soil bacteria, fungal, and arbuscular mycorrhizae (AM) fungal communities to continuous sugarcane cultivation has not been thoroughly documented. Using MiSeq sequencing technology, we analyzed soil samples from sugarcane fields with 1, 10, and 30 years of continuous cropping to see how monoculture time affected sugarcane yield, its rhizosphere soil characteristics and microbiota. The results showed that continuous sugarcane planting reduced sugarcane quality and yield. Continuous sugarcane planting for 30 years resulted in soil acidification, as well as C/N, alkali hydrolyzable nitrogen, organic matter, and total sulfur content significantly lower than in newly planted fields. Continuous sugarcane planting affected soil bacterial, fungal, and AM fungal communities, according to PCoA and ANOSIM analysis. Redundancy analysis (RDA) results showed that bacterial, fungal, and AM fungal community composition were strongly associated with soil properties and attributes, e.g., soil AN, OM, and TS were critical environmental factors in transforming the bacterial community. The LEfSe analysis revealed bacterial families (e.g., Gaiellaceae, Pseudomonadaceae, Micromonosporaceae, Nitrosomonadaceae, and Methyloligellaceae) were more prevalent in the newly planted field than in continuously cultivated fields (10 and 30 years), whereas Sphingomonadaceae, Coleofasciculaceae, and Oxyphotobacteria were depleted. Concerning fungal families, the newly planted field was more dominated than the continuously planted field (30 years) with Mrakiaceae and Ceratocystidaceae, whereas Piskurozymaceae, Trimorphomycetaceae, Lachnocladiaceae, and Stigmatodisc were significantly enriched in the continuously planted fields (10 and 30 years). Regarding AMF families, Diversisporaceae was considerably depleted in continuously planted fields (10 and 30 years) compared to the newly planted field. These changes in microbial composition may ultimately lead to a decrease in sugarcane yield and quality in the monoculture system, which provides a theoretical basis for the obstruction mechanism of the continuous sugarcane planting system. However, continuous planting obstacles remain uncertain and further need to be coupled with root exudates, soil metabolomics, proteomics, nematodes, and other exploratory methods.

## 1. Introduction

Sugarcane is a globally important crop since it contributes to sustainable energy and sugar production [1,2]. China is the world’s third-largest sugar producer, and sugarcane has accounted for more than 90% of total sugar production in recent decades. Sugarcane is often planted as a monoculture in China’s subtropical and tropical regions [3,4]. Ratoon sugarcane is a regenerative crop comprised of germinated buds from previous sugarcane stubble, which reduces planting materials and tillage costs and matures earlier than newly planted sugarcane [5]. On the other hand, continuous monoculture severely inhibits sugarcane growth and yield by causing soil degradation and triggering soil-borne diseases [4,6], resulting in a drastic economic loss to the Chinese sugarcane industry [7]. This general phenomenon has also been observed in both perennial and annual crops, such as melons [8], soybeans [9], bananas [10], coffee [11], and tea [12]. Consecutive monoculture obstacles are commonly attributed to the accumulation of soil-borne pathogens or autotoxic substances and to the deterioration of physiochemical soil properties [4,13,14]. A great deal of research has revealed that shifts in the soil microbiome, such as the proliferation of bacterial and fungal pathogens, are the leading causes of consecutive monoculture obstacles [15,16]. For example, Song et al. [15] showed that Coptis Chinensis monoculture increased soil-borne pathogens, such as *Fusarium,* which caused root rot disease and reduced crop yield. Similarly, potato monoculture increased soil-borne pathogens, such as *Fusarium*, which increased disease incidence and reduced crop yield [17].

Under continuous sugarcane cropping systems, there is a limited understanding of the shifts in physiochemical soil characteristics and microbial communities [18]. Hence, long-term sugarcane test fields may be helpful to provide us with comprehensive information about the impacts of sugarcane-grown fields with different years of monoculture history on soil fertility and ecosystem functions. Thus, we hypothesized that sugarcane monoculture would affect physiochemical soil properties and microbial communities, thereby reducing sugarcane production. We used taxonomy analysis to investigate the microbial communities in sugarcane-grown fields with 1, 10, and 30 years of monoculture history in Fujian Province, China. The objectives of this research were to (i) estimate the sugarcane yield, physiochemical soil parameters and soil microbial diversity, structural and compositional shifts related with the different consecutive monoculture histories of the sugarcane fields, (ii) investigate the associations between the dominant soil microbial taxa and physiochemical soil parameters of sugarcane monoculture fields, and (iii) consider how changes in microbial communities caused by long-term monoculture may affect agronomic sugarcane parameters.

## 2. Materials and Methods

### 2.1. Location Description and Sample Collection

The study location is at Wanqian Village, Songxi County, Nanping City, Fujian Province, China (27°43′N, 118°72′E). The annual temperature and average yearly precipitation are 19 °C and 1650 mm, respectively. The soil is sandy loam (clay 3.87%, silt 22.80% and sand 73.33%). Soil samples were collected from three sugarcane fields, established in the early spring of 2017, 2008, and 1988 (labeled as “NCC”, “CC10” and “CC30”, respectively), with a distance of less than 150 meters between all fields. The three time-series fields had consistent agronomic management, soil type, and fertilization regimes. Each sugarcane growing trial site had three replications, with sugarcane rows spaced 1.4 m apart and plot areas varying from 30.6 to 50.4 m^2^. All plots were fertilized with 300 kg/hm^2^ of urea, 75 kg/hm^2^ of K_2_O, and 300 kg/hm^2^ of calcium superphosphate each season, as is customary in the area. Thirty and seventy percent of total fertilizer applications were applied to sugarcane in the seedling and elongation periods. On 28 December 2017, agronomic sugarcane traits were evaluated during the sugarcane maturation stage, and five rhizosphere soil samples were obtained from each plot using the S-sampling method and blended as a biological replicate. Individual soil samples were packaged in sterile plastic bags and transferred to the laboratory in an icebox. All soil samples were divided into two parts after passing through a 2 mm sieve. Afterward, a portion of each sample was air-dried to evaluate the physicochemical soil attributes, while the remainder was stored at −80 °C for DNA extraction.

### 2.2. Measurement of Sucrose Content and Theoretical Yield 

Thirty sugarcane plants were randomly selected from each plot and the stalk height was measured using a measuring tape; the diameter was determined using a Vernier caliper. An Extech Portable Sucrose Brix Refractometer (Mid-State Instruments, CA, USA) was used to determine sucrose content, which was calculated using the formula: sucrose (%) = brix (%) × 1.0825 − 7.703 [6]. The following equations were used to calculate theoretical sugarcane production [6]: (a) single stalk weight (kg) = (stalk diameter (cm))^2^ × (stalk height (cm) − 30) × 1 (g/cm^3^) × 0.7854/1000; and (b) theoretical production (kg/hm^2^) = single stalk weight (kg) × productive stem numbers (hm^2^).

### 2.3. Measurement of Soil Chemical Properties

Soil suspensions with water (1:2.5 WV^−1^) were prepared to estimate soil pH using a pH meter (PHS-3C, INESA Scientific Instrument Co., Ltd, Shanghai, China) [19]. An elemental analyzer was used to measure total sulfur (TS), total carbon (TC), and total nitrogen (TN) in the soil extracts (Thermo Scientific™, Waltham, MA, USA). The Molybdenum Blue procedure was used to assess available phosphorus (AP) using hydrochloric acid and ammonium fluoride [20]. The alkaline hydrolyzable diffusion method [21] and the potassium dichromate external heating method [22] were used to determine available nitrogen (AN) and organic matter (OM). Whereas available potassium (AK) was extracted using ammonium acetate and quantified using flame photometry [23]. Total potassium (TK) and total phosphorus (TP) levels were determined by first digesting the soil using the H_2_SO_4_-HClO_4_ procedure and then calculating the levels, as described for AP and AK.

### 2.4. Soil DNA Extraction and PCR Amplification

DNA was extracted from each soil sample using a Power Soil DNA Isolation Kit (MoBio Laboratories Inc., Carlsbad, USA) as per the manufacturer’s directions. A NanoDrop 2000 spectrophotometer (Thermo Scientific, Waltham, MA, USA) was used to determine soil DNA purity and concentration. To amplify 16S rRNA and 18S rRNA gene segments, the primers 338F/806R [24,25] and SSU0817F/SSU1196R [26] were used. PCR conditions were (95 °C for 3 min, followed by 35 cycles of 95 °C for 30 s, 55 °C for 30 s, and 72 °C for 45 s, with a final extension at 72 °C for 10 min) (GeneAmp 9700, ABI, Vrmon, CA, USA). PCR reactions were carried out in triplicate in a 20-μL mixture with 2 μL of 2.5 mM deoxyribonucleoside triphosphate (dNTPs), 4 μL of 5 × Fast Pfu buffer, 0.4 μL of Fast Pfu polymerase, 0.4 μL of every primer (5 μM), and template DNA (10 ng). Other DNA samples served as templates for nested PCR in AMF special primers. The first round of PCR was performed using AML1F/AML2R [27], while the second round was conducted with AMV4.5NF/AMDGR [28]. The amplification conditions were the same as before [29].

### 2.5. Illumina MiSeq Sequencing

Amplicons were extracted using the AxyPrep DNA Gel Extraction Kit (Axygen Biosciences, Union City, CA, USA). Then, QuantiFluor^™^-ST (Promega, Madison, WI, USA) was used for quantification. According to the standard protocols, purified amplicons were pooled in equimolar and paired-end sequences on an Illumina MiSeq platform (Majorbio, Shanghai, China).

### 2.6. Processing and Analyzing of Sequencing Data

The 16S rRNA gene sequences were processed using QIIME v.1.9.1 [30], USEARCH v.7.0 [31], and in-house scripts [32]. The quality of the paired-end Illumina reads was checked by Fastp v.0.19.6 [33] and processed in the following steps with USEARCH: joining of paired-end reads and relabeling sequencing names; removal of barcodes and primers; filtering of low-quality reads, and finding non-redundancy reads. Unique reads were clustered into OTUs with 97% similarity. The representative sequences were picked by UPARSE v.7.0.1090 [34]. OTUs were aligned to the SILVA 132 database to remove sequences from chimera and host plastids [35]. USEARCH was used to produce the OTU table, and the taxonomy of the representative sequences was classified using the RDP classifier v.2.11 [36]. 

The richness, such as ACE, number of observed OTUs, and diversity (Shannon indexes) [37], was utilized for estimating the diversity of microbial communities in every soil sample using the Mothur pipeline [38]. Nonparametric statistics based on the Bray–Curtis dissimilarity index were performed on the OTU data to quantify the observed difference better. To determine whether there was a significant difference in microbial community composition among different sugarcane monoculture times, principal coordinate (PCoA) analysis and an analysis of similarities (ANOSIM) were performed [39]. Wilcoxon rank-sum tests based on OTUs were used to analyze the differences in OTU abundance and taxonomy, and the corresponding *P* values were corrected for multiple testing using an FDR of 0.05 [40]. Linear discriminant analysis effect size (LEfSe) was used to identify significant differences in microbial taxa between groups. In LEfSe analysis, the Kruskal-Wallis (KW) sum-rank test was used to find features with significantly different abundances between assigned classes, and then linear discriminant analysis (LDA) was used to quantify the effect size of each feature [41]. R (version 4.0.2) was used to conduct a redundancy analysis (RDA) to investigate the influence of soil physiochemical parameters on bacterial, fungal, and AMF abundance at the phylum level. The Mantel test investigated the relationship between diverse environmental conditions and microbial community structure [42]. Pearson’s correlation coefficients across microbial taxa at the genus level, sugarcane monoculture time, and soil characteristics were calculated using R (4.0.2) and then visualized using Cytoscape software (v3.6.1). Soil physiochemical and agronomic attributes across the time-series of sugarcane field samples were compared using LSD test (*p* = 0.05) in DPS software (v.7.05).

## 3. Results

### 3.1. Sucrose Content and Theoretical Yield

The agronomic attributes, sugar content and theoretical production of the sugarcane of three time-series sugarcane fields are shown in Table 1. Overall, agronomic sugarcane traits in “CC10” and “CC30” were lower than those in “NCC”. The stalk height, single stalk weight, sucrose content and theoretical yield of sugarcane plants was significantly lower in “CC30” than in “NCC”. Furthermore, there was no significant difference in available stalk across all continuous cropping fields.

### 3.2. Soil Physiochemical Properties of Continuous Sugarcane Fields

The physiochemical attributes of soil during continuous cropping of sugarcane are shown in Table 2. Overall, soil pH, OM, TN, TS, TK, C/N, and AN decreased as the sugarcane planting period increased, while TP, AK increased. The pH, C/N, OM, TS, and AN contents of "CC30" soil were significantly lower than those of "NCC" soil. There was no significant difference in soil TN, TP, TK, AP, and AK between all continuous cropping soils. Pearson correlation analysis showed that there was a significant negative correlation between planting years and nutrients (such as TS, OM, pH, AN, and C/N ratio) (r^2^ < −0.6, *p* < 0.05). OM was strongly correlated (r^2^ > 0.76, *p* < 0.01) with AN, pH, and C/N ratio. pH was strongly correlated (r^2^ > 0.72, *p* < 0.05) with AN and C/N ratio (Figure 5).

### 3.3. Microbial Alpha and Beta Diversity

**Figure 1:**
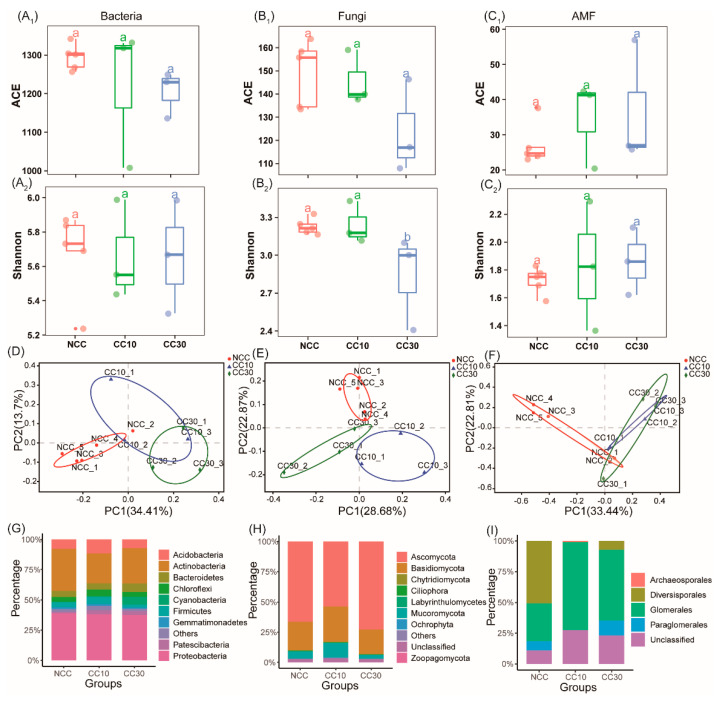
Box plots showing ACE richness and Shannon diversity index of bacteria, fungi and AM fungi (**A1**–**C2**). Unconstrained PCoA (for principal coordinates PC1 and PC2) with Bray-Curtis distance from bacterial (**D**), fungal (**E**) and arbuscular mycorrhizal fungal (**F**). Relative abundances of the top bacterial (**G**), fungal (**H**), and arbuscular mycorrhizal fungal (**I**) phyla or Orders. NCC: first-year monoculture; CC10: ten-year continuous monoculture; CC30: thirty-year continuous monoculture.

According to rarefaction analysis, our population captured the most microbiota members from each soil sample (Figure S1). All samples generated 462,349, 522,802 and 238,846 high-quality 16S rRNA, 18S rRNA, and AMF gene (Table S1). We discovered that the composition of the soil’s bacterial, fungal, and arbuscular mycorrhizae fungal microbial communities differed depending on the sugarcane cropping history. Unconstrained principal coordinate analysis (PCoA) of Bray-Curtis distance and ANOSIM results revealed that continuous sugarcane cropping history influenced the bacterial, fungal, and AMF community similarity distance (Figure 1 and Table 3). PCoA analysis showed different patterns of fungal, bacterial, and AMF communities in sugarcane fields continuously cropped for different years, with the first two axes illustrating 48.11%, 51.55%, and 56.25% of the total shift in microbial data, respectively. Additionally, bacterial, fungal, and AMF communities in 1-year (NCC) soils samples were separated from the CC10 and CC30 soil samples (Figure 1D–F). The ANOSIM analysis revealed that the R values of NCC and other continuous soils were greater than 0.5, with NCC and CC30 in the bacteria reaching 0.86. In bacteria or fungi, however, the R-value between CC10 and CC30 is less than 0.5. These findings indicate that NCC’s soil microbial population variation was greater than that of continuous soils (CC10 and CC30), while the difference between CC10 and CC30 was small. Figure 1A,C depicts the alpha diversity of soils under continuous sugarcane cropping. In “CC10” and “CC30” sugarcane fields, the number of observed OTUs and ACE indexes of both fungal and bacterial communities decreased when compared to sugarcane fields “NCC.” Similarly, the Shannon diversity index showed consistent richness, whereas the AMF community showed the opposite result. These findings confirmed that, as sugarcane planting time increased with the same management practices, fungal and bacterial species richness and diversity decreased while AM fungi increased.

### 3.4. Microbial Community Composition under Continuous Sugarcane Cropping

The relative abundances of bacteria and fungi were examined at the phylum and order level to investigate their response to a continuous monoculture of sugarcane with varying cropping histories. In all samples, dominant fungal and bacterial phyla and orders were found in three sugarcane fields consecutively cropped for different years. Relative abundance of Proteobacteria was high across all the samples, followed by Actinobacteria, Acidobacteria, Bacteroidetes, Chloroflexi, Firmicutes, Patescibacteria, Cyanobacteria, Gemmatimonadetes; these were the dominant bacterial phyla (Figure 1G). The relative abundances of Proteobacteria decreased with the increase of consecutive planting years. While, the abundances of Bacteroidetes, Patescibacteria and Cyanobacteria increased. Turning to the fungal community, three dominant fungal phyla, Ascomycota, Basidiomycota and Mucoromycota, and AM fungal orders, such as Glomerales and Diversisporales, were detected in all soil samples (Figure 1H,I). The relative abundances of the Diversisporales decreased with an increase in consecutive planting years, while, the abundances of Glomerales showed a reverse trend.

### 3.5. Analysis of Soil Microbial Differences

**Figure 2:**
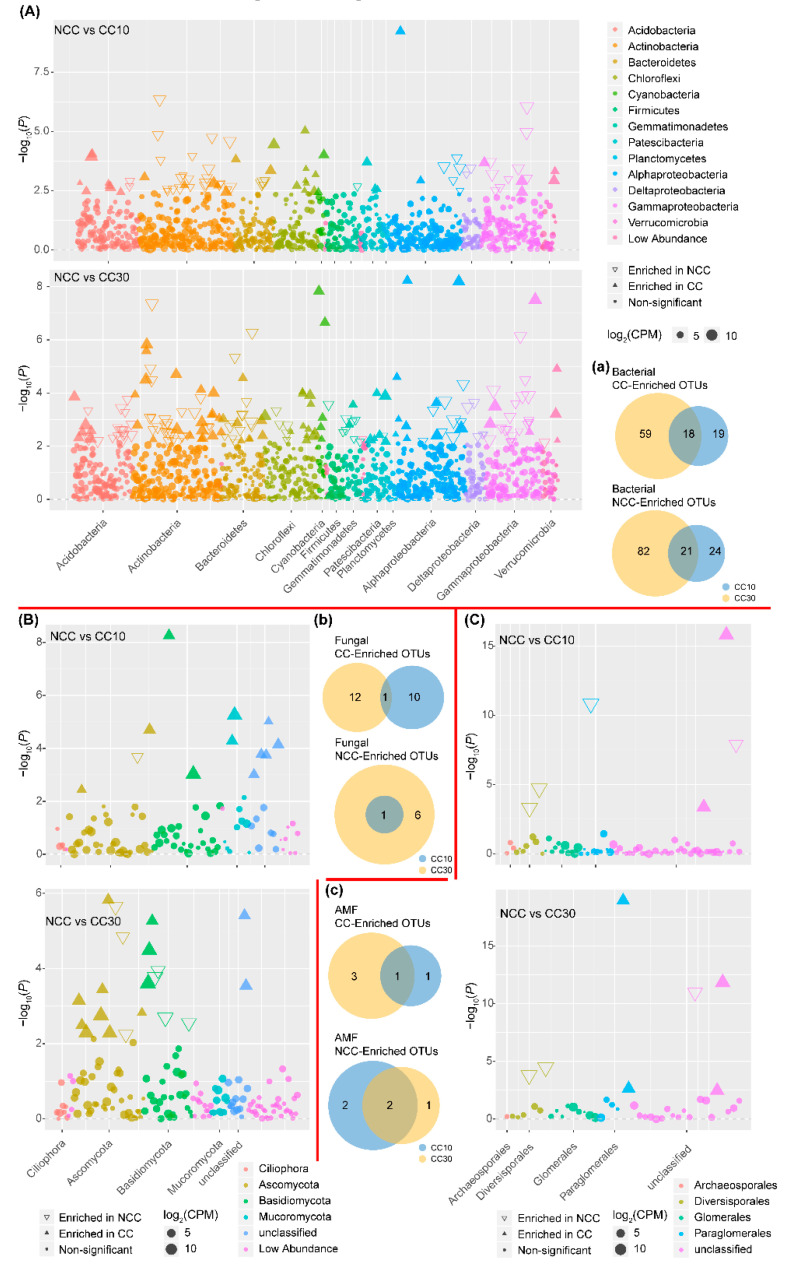
Taxonomic characteristics of differential bacteria (**A**), fungal (**B**) and AMF (**C**) between the “NCC” and “CC” groups microbiota. OTUs enriched in the “NCC” or “CC” groups are represented by filled or empty triangles, respectively (FDR adjusted *p* < 0.05, Wilcoxon rank sum test). OTUs are arranged in taxonomic order and colored according to the phylum or, for Proteobacteria, the class. CPM, counts per million. Among the three types of a, b, and c microorganisms, the overlapping part is rich or scarce in CC10 and CC30.

To determine soil microbial differences in sugarcane fields with different cropping years, we used DESeq2 to compare NCC and CC10, NCC and CC30 microbial OTUs and visualized the results using a Manhattan plot. The results showed that NCC vs. CC10 and NCC vs. CC30 had OTU differences for the majority of the bacterial levels. However, OTUs associated with Actinobacteria, Actinobacteria and Proteobacteria were greatly different. There were 180 OTUs with significant differences between CC30 and NCC, in which 77 OTUs were enriched in CC30. There were 82 OTUs with substantial differences between CC10 and NCC, 37 of them were enriched in CC10 (Figure 2A). According to Veen’s graph, 18 bacterial OTUs were co-enriched by CC10 and CC30, and 21 OTUs are reduced together (Figure 2a).

Differences in OTUs between NCC and CC were linked to Ascomycota, Basidiomycota, and Mucoromycota. There were 20 OTUs with a significant difference between CC30 and NCC, with 13 of them being enriched in CC30. There were 12 OTUs with significant differences between CC10 and NCC, 11 of which were enriched in CC10 (Figure 2B). According to Veen’s observations, one fungus OTU was co-enriched by CC10 and CC30, and one OTU was reduced by one (Figure 2b). There was a difference in OTUs associated with Diversisporales and Paraglomerales between NCC and CC in the AM fungi. The number of OTUs with a significant difference between CC30 and NCC was seven, with four of them being enriched in CC30. The number of OTUs in CC10 that differed significantly from NCC was 6, of which 2 were enriched in CC10 (Figure 2C). According to Veen’s findings, one AM fungus OTU was co-enriched in CC10 and CC30, and two OTUs were reduced together (Figure 2c).

**Figure 3:**
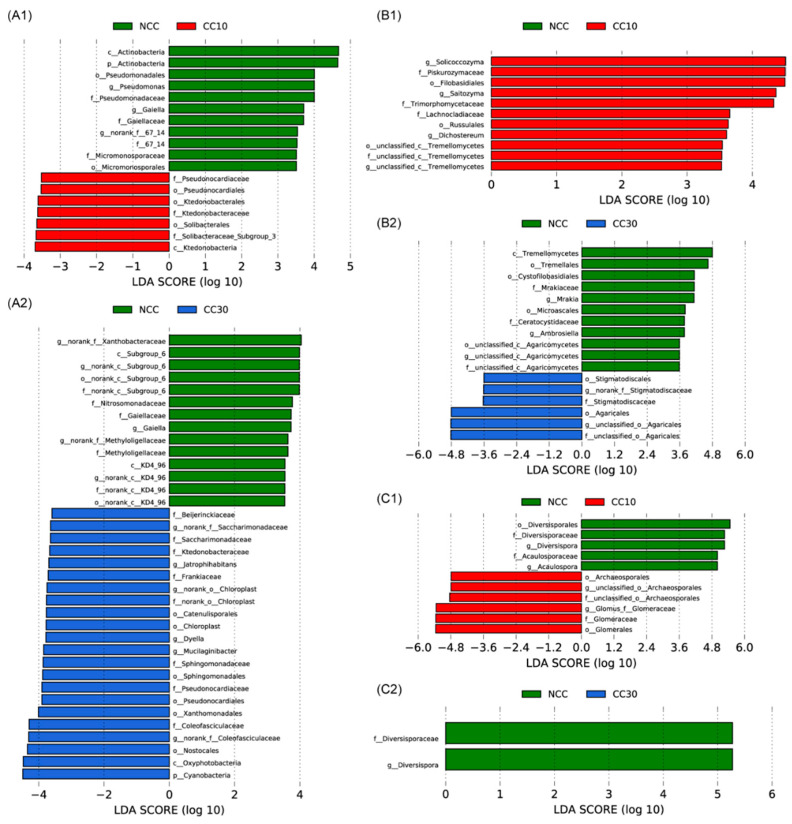
Liner discriminant analysis, in conjunction with effect size measurements, identifies the differentially abundant taxa between cultivation years: NCC vs. CC10/CC30 of bacterial (**A1**,**A2**), fungal (**B1**,**B2**), and AM fungal taxa (**C1**,**C2**). Lineages with LDA values greater than 3.5 are shown.

LEfSe analysis (LDA > 3.5) revealed that soil bacteria, fungi, and arbuscular mycorrhizae fungi changed across sugarcane cultivation years. At the bacterial phylum level, Actinobacteria was significantly depleted in the “CC10” field compared to the “NCC” field, while Cyanobacteria was greatly enriched in the "CC30" field (Figure 3A). At the bacterial family level, the “NCC” field was more dominant than "CC10" or "CC30" with Gaiellaceae, Pseudomonadaceae, Micromonosporaceae, Nitrosomonadaceae, and Methyloligellaceae. Pseudonocardiaceae, Ktedonobacteraceae, Beijerinckiaceae, Saccharimonadaceae, Frankiaceae, Sphingomonadaceae, Coleofasciculaceae, and Oxyphotobacteria, on the other hand, were depleted. The top fungal phyla, such as Ascomycota and Basidiomycota, were found in the fields “NCC”, “CC10”, and “CC30”. The “NCC” field was more dominant at the fungal family level than "CC30" with Mrakiaceae and Ceratocystidaceae. Piskurozymaceae, Trimorphomycetaceae, Lachnocladiaceae, and Stigmatodiscaceae were significantly enriched in the “CC10” or “CC30” field (Figure 3B). Glomerales and Diversisporales were the top arbuscular mycorrhizae fungi orders. At the arbuscular mycorrhizae fungal family level, the Diversisporaceae was depleted considerably in the “CC10” and “CC30” fields compared to the “NCC” field, whereas the Glomeraceae was significantly enriched in the “CC10” field (Figure 3A).

### 3.6. Influence of Soil Physiochemical Parameters on Soil Microbial Composition

**Figure 4:**
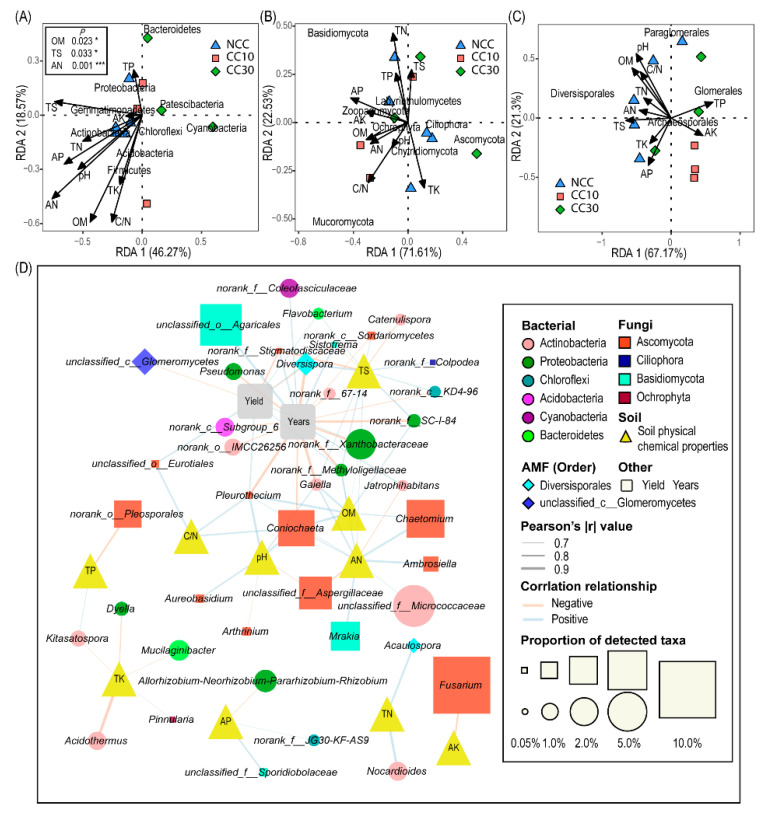
Redundancy analysis (RDA) identified nine selected ecological variables for shaping bacterial (**A**), fungal (**B**) and AM fungal (**C**) communities. Correlation analysis between microbial genus or order level and soil nutrients (**D**). TN, total nitrogen; TK, total potassium; TP, total phosphorus; TS, total sulfur; OM, organic matter; AN, available nitrogen; AK, available potassium; AP, available phosphorus; C/N, C:N ratio; NCC: first-year monoculture; CC10: ten-year continuous monoculture; CC30: thirty-year continuous monoculture.

Redundancy analysis (RDA) showed different patterns of microbial communities by soil physiochemical properties, with the first two axes explaining 64.84%, 94.14%, and 88.47% of the total shift in the bacterial, fungal and arbuscular mycorrhizae fungal data, respectively (Figure 4A–C). Proteobacteria were negatively correlated to C/N and OM, while positively related to TP; Actinobacteria, Acidobacteria and Firmicutes were positively correlated to AP, TN, AN, and pH; and Cyanobacteria were negatively correlated to TS. Soil AN, OM, and TS content were key environmental factors that drive bacterial communities’ composition at the phylum level. *Ascomycota* was negatively correlated with AP, AK and OM; Basidiomycota was negatively related to TK, while positively related to TN and TP. For arbuscular mycorrhizae fungi, Diversisporales were positively correlated to AN, TS, and TN, while negatively associated with AK; Glomerales were positively correlated to TP, while negatively related to TS, TK, and AN.

Furthermore, we correlated cropping times, soil attributes, and microbial taxa, and the results revealed that soil nutrients could influence microorganism abundance at the genus level. Soil AN content and *Ambrosiella*, *Chaetomium*, *Coniochaeta*, *Mrakia*, *Pleurothecium*, and *Gaiella* abundances showed a positive correlation. Furthermore, soil AP content was negatively correlated with *Allorhizobium-Neorhizobium-Pararhizobium-Rhizobium*, while soil AK content was negatively correlated with *Fusarium*. Soil pH and OM content were positively correlated with *Pleurothecium*, *Coniochaeta*, *Gaiella,* while soil TS content was positively correlated with *Gaiella*, *Flavobacterium* and *Diversispora*, and negatively correlated with *Sistotrema* and *Catenulispora* (Figure 4D, Table S2).

**Figure 5:**
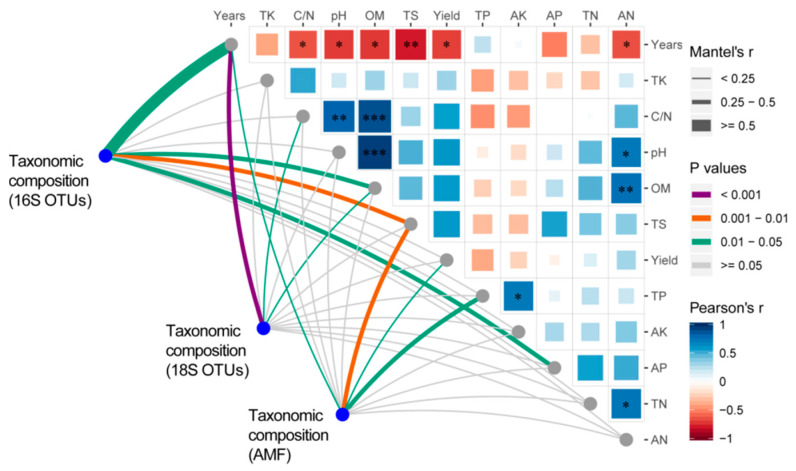
The relationship between environmental variables and soil microbial community composition. Pairwise comparison of ecological factors and color gradients representing Pearson’s correlation coefficient. Taxonomic groups were related to each ecological aspect using the Mantel test. Edge width corresponds to the Mantel’s r statistic for the corresponding distance correlations, and edge color denotes the statistical significance based on 999 permutations. TN, total nitrogen; TK, total potassium; TP, total phosphorus; TS, total sulfur; OM, organic matter; AN, available nitrogen; AK, available potassium; AP, available phosphorus; C/N, carbon–nitrogen ratio. * *p* < 0.05. ** *p* < 0.01. *** *p* < 0.001.

We correlated distance-corrected dissimilarities of taxonomic community composition with environmental factors to identify the main ecological drivers that influence the composition of microbial communities (Figure 5). Overall, Mantel test analysis revealed that the sugarcane planting years significantly impacted the bacterial community (r = 0.55, *p* < 0.05). Furthermore, soil nutrients, such as TS, AP, and OM, influence bacterial communities. The sugarcane cultivation years were also the main driving factor in the fungal community composition (r = 0.44, *p* < 0.001), and soil C/N and OM had significant effects on its community. While TS (r = 0.35, *p* < 0.01), TP, and cropping years also significantly affected AM fungal communities.

## 4. Discussion

In this work, we examined soil samples from sugarcane fields with 1, 10, and 30 years of continuous cropping history to determine the influence of monoculture on edaphic characteristics and soil microbiota, thereby influencing sugarcane productivity. Continuous sugarcane cropping hinders plant growth and decreases sugarcane biomass and yields [6,43]. This study confirmed that sugarcane fields with 10 and 30-years of monoculture history decreased the stalk diameter, single stalk weight, available stalk number per hectare, and theoretical yield of sugarcane plant (Table 1), which is in agreement with previous findings [43]. This general phenomenon also occurred in both perennials and annual crops, such as soybean [9], potato [44], coffee [11], and tea [45], of which growth was seriously hindered in a continuous monoculture system. Continuous sugarcane monoculture reduced soil fertility and caused soil degradation in sugarcane fields [4,6,43], resulting in lower theoretical sugarcane output in CC10 and CC30 fields than in the NCC field. 

Physiochemical characteristics of the soil are extensively known as being crucial to the sustainability of agricultural production systems. In the present study, continuous cropping of sugarcane decreased the soil quality indicators of CC10 and CC30 fields than in the NCC field. Sugarcane fields (CC10 and CC30) declined the soil pH and OM due to the long-term use of acid-forming fertilizers (e.g., urea) rather than organic fertilizer. Similarly, Hartemink et al. [46] also revealed that acid input as ammonium-N fertilizers and alkali removal as uptake of ammonium-N by the plant could lead to soil acidification. Additionally, soil acidification in sugarcane fields may be attributed to the significant reduction of bases through harvested sugarcane and the leaching of the cations [47,48]. This obstacle has also been observed in other sugarcane-producing countries like Papua New Guinea, Australia, Fiji, and China [4]. Sugarcane plants efficiently utilized the AN and AP content of the soil, which could decrease the AN and AP content in CC10 and CC30 fields, which is in agreement with previous reports that continuous cropping of sugarcane leads to a reduction in soil macronutrients, such as AN and AP [18,49,50,51]. Naranjo et al. [50] found that sugarcane monoculture for 30 years decreased the total soil OM and TN by 21% and 37%, respectively, which agrees with our results. Reddy et al. [52] found that soil TS reduced by continuous planting for 27 years by 18.13%. In addition, accumulation of allelochemicals discharged from sugarcane root exudation and debris, phytotoxic microflora development and one-sided nutrient exhaust cause soil sickness under continuous monoculture of sugarcane [53]. In conclusion, soil acidification due to mineral fertilizers decreased soil OM or accumulated sugarcane residue and allelochemicals could be significant factors for reducing the production of sugarcane fields with 10 and 30-years of monoculture history.

A great deal of research showed that soil microbiota has an essential role in maintaining soil function, quality and ecosystem sustainability [43,54]. Analysis of soil microbial community variation under consecutive monoculture systems can help understand the low yield of sugarcane associated with continuous cropping. This research demonstrated that sugarcane fields with 10 and 30 years of monoculture history declined the richness of microbiota, which was consistent with the previous findings [6]. Similarly, the abundance and diversity of fungi declined with the increasing years of long-term monoculture of coffee and *Panax notoginseng,* respectively [11,55]. One of the leading threats to ecosystem function is the loss of microbial diversity [56]. Consequently, the reduction in soil microbial species richness could decrease microbial taxa that promote plant growth or inhibit plant disease [57], reducing the sugarcane growth and yield of sugarcane fields with 10 and 30-years of monoculture history.

In this study, PCoA and ANOSIM analysis showed that continuous cropping of sugarcane influenced the bacterial, fungal and AM fungal communities (Figure 2A,B), which is consistent with of Chen et al. [58] in which continuous cropping of peanuts with different ages also significantly affected the variation of microbial community structures. This phenomenon was also noticed in vanilla [59], *Panax notoginseng* [55], coffee [11], and tea [12] continuous cropping systems.

According to the analysis results of Deseq2 and LEfSe, we know that continuous cropping changes the composition of soil microbial communities, including soil bacteria, fungi, and AM fungi, and that these microorganisms are closely related to soil functions. At the bacteria phylum level, we found that the continuous cultivation of sugarcane resulted in a significant decrease in Actinobacteria and a significant increase in Cyanobacteria. Members associated with Actinobacteria are potential biocontrol agents and can interact with plants and promote their growth [60,61]. Consecutive monoculture of cotton decreased the OM of soil, which led to a decline in Actinobacterial population [62,63]. Arafat et al. [12] found that a 30-year-old tea plantation had significantly lower soil pH, which in turn decreased the abundance of Chloroflexi and Actinobacteria. Members associated with Cyanobacteria are the producers of toxins such as neurotoxins or hepatotoxins; these toxins are related to the morbidity and mortality of various human diseases, aquatic mammals and fish [64]. A previous study found that long-term monoculture soybean significantly increased Cyanobacteria abundance in topsoil [65].

At the bacterial family level, continuous cropping of sugarcane reduced the beneficial microbial taxa over time, such as Gaiellaceae, Pseudomonadaceae, Micromonosporaceae, and Nitrosomonadaceae (Figure 3A). Previous investigations have determined that Gaiellaceae are related to the carbon and nitrogen cycle [66]. Wu et al. [67] have found that the family Pseudomonadaceae decreased under consecutive monoculture. Members associated with Micromonosporaceae can degrade chitin, cellulose, lignin, and pectin, and these microorganisms play an essential role in the turnover of organic plant material [68], while Nitrosomonadaceae is related to the nitrogen cycle [69,70]. 

Tayyab et al. [4] observed that sugarcane monoculture elevated soil acidity and degradation, which not only increased some fungal taxa but also decreased it by increasing cropping time. Similarly in this study, continuous cropping of sugarcane not only reduced microbial taxa but also enriched the microbial taxa over time (Figure 3B). At the AM fungal family level, continuous cropping of sugarcane reduced the microbial taxa over time, such as Diversisporaceae and Acaulosporaceae (Figure 3C). Xiang et al. [71] found that both Diversisporaceae and Acaulosporaceae were positively correlated with herbaceous and shrubby biomass, and these results are consistent with our results. 

The results of redundancy analysis (RDA) and Pearson’s correlation analysis revealed that bacterial, fungal and AM fungal community compositions were closely associated with soil properties, which suggests that the impacts of continuous cropping on the soil microbial community are linked to the alteration of soil chemical properties (Figure 4A–C) [57]. On the contrary, microbial changes in a continuous monoculture of sugarcane systems are due to the shifts in soil chemical properties and may be attributed to the long-term influence of sugarcane plant residues or root exudates [72,73]. On the other hand, mental test analysis showed that cultivation years, total soil sulfur, available phosphorus, organic matter and pH value are the main factors affecting the composition of soil microbial communities [4].

The sugarcane fields (1, 10 and 30 years old) were continuously cropped with similar agronomic practices; hence we carried out the correlation among the consecutive monoculture time with microbial taxa (at phylum and genus level) of these fields (Tables S3 and S4, Figure 4D). Under connective monoculture of sugarcane, the relative abundances of Proteobacteria, Actinobacteria, Acidobacteria, Chloroflexi, Firmicutes, Basidiomycota, Gemmatimonadetes, and Verrucomicrobia declined in overtime (Table S3) and this is consistent with previous findings [12,59]. Proteobacteria can enhance disease resistance in plants and can promote plant growth [74]. Members associated with Firmicutes can suppress soil-borne diseases [75,76], promote plant growth, and enhance drought tolerance in various plants [77,78,79,80]. Multiple studies have demonstrated that Firmicutes and Basidiomycota were less abundant in disease conducive soils than in suppressive soils [59,74]. Members associated with Actinobacteria are potential biocontrol agents and can interact with plants and promote their growth [60]. Consecutive monoculture of cotton decreased the OM of soil, which led to a decline in Actinobacterial population [62,63]. Arafat et al. [12] found that the 30-year-old tea plantation significantly lowered the soil pH, which in turn decreased the abundance of Chloroflexi and Actinobacteria. The data obtained from Illumina sequencing revealed some unique microbial taxa, either beneficial or pathogenic, that were reduced or increased in the three-time series sugarcane field. Depending on their role in another ecosystem [81,82], we can also speculate how these taxa respond to continuous sugarcane cropping. Therefore, our research may grant us a way to describe the effect of continuous monoculture on the abundance of beneficial and pathogenic microbial taxa that reduce sugarcane yield. Continuous sugarcane cropping reduced the beneficial microbial taxa at the genus level, such as *Bacillus*, *Streptomyces*, and *Talaromyces* (Table S4). Previous investigations have determined that *Bacillus* sp. can suppress soil-borne diseases [75,76], promote plant growth and enhance drought tolerance in various plants such as rice [77], *Brachypodium* sp. [78], pepper [79], and *Arabidopsis* sp. [80]. Likewise, *Streptomyces* is a well-studied genus that is economically important in agricultural systems as it contains various potential biocontrol agents [83]. Species of *Talaromyces* genus are important fungal antagonists used as a bio-control agent of soil-borne pathogens such as *Verticillium dahlia* and *Fusarium oxysporum* [84,85,86]. These results demonstrated that declined sugarcane yield in 10- and 30-year-old fields might be associated to a decline in potentially beneficial microbes, reduction in soil pH and soil fertility.

## 5. Conclusions

Overall, our results demonstrated that low sugarcane production under the long-term continuous cropping system might be associated with changes in microbial communities and soil physiochemical features, such as declines in microbial diversity and potentially beneficial microbes, reductions in soil pH, and soil fertility. More studies are required to further disentangle the triangle associations among sugarcane yield, soil characteristics, and potentially pathogenic microbiota. This research grants us an invaluable avenue for progressing sustainable agricultural measures to enhance microbial activity and boost sugarcane production in continuous cropping soils, which is essential for sugarcane production in China.

## Figures and Tables

**Table 1:** Sugarcane plant agronomic characteristics, sugar content, and theoretical production.

Sugarcane Fields	Sucrose Content (%)	Available Stalk Number (hm^−2^)	Stalk Height (cm)	Stalk Diameter (cm)	Single Stalk Weight (kg)	Theoretical Production (kg/hm^2^)
NCC	10.29 ± 0.55 ^ab^	90,667 ± 3559 ^a^	256.0 ± 4.0 ^a^	1.71 ± 0.02 ^a^	0.52 ± 0.01 ^a^	47,043 ± 1051 ^a^
CC10	11.53 ± 0.32 ^a^	91,111 ± 2940 ^a^	238.3 ± 4.4 ^ab^	1.67 ± 0.02 ^a^	0.46 ± 0.02 ^ab^	41,809 ± 2033 ^ab^
CC30	08.17 ± 0.85 ^b^	89,556 ± 2320 ^a^	236.7 ± 8.8 ^b^	1.63 ± 0.04 ^a^	0.44 ± 0.04 ^b^	38,921 ± 3229 ^b^

Values followed by different lowercase letters within the same column show significant differences (LSD test, *P* < 0.05). NCC: first-year monoculture; CC10: ten-year continuous monoculture; CC30: thirty-year continuous monoculture.

**Table 2:** Soil physiochemical characteristics.

	Units	NCC	CC10	CC30
pH		5.73 ± 0.14 ^a^	4.85 ± 0.22 ^b^	4.78 ± 0.14 ^b^
OM	g/kg	31.57 ± 4.48 ^a^	19.26 ± 5.24 ^ab^	15.42 ± 2.56 ^b^
TN	g/kg	1.02 ± 0.09 ^a^	0.90 ± 0.05 ^a^	0.90 ± 0.12 ^a^
TS	g/kg	0.27 ± 0.00 ^a^	0.23 ± 0.02 ^b^	0.21 ± 0.02 ^b^
TP	g/kg	0.46 ± 0.07 ^a^	0.57 ± 0.04 ^a^	0.54 ± 0.11 ^a^
TK	g/kg	25.59 ± 1.11 ^a^	25.38 ± 1.45 ^a^	23.57 ± 0.84 ^a^
C/N		31.18 ± 1.78 ^a^	21.18 ± 5.16 ^ab^	18.10 ± 4.74 ^b^
AN	mg/kg	100.53 ± 9.46 ^a^	87.05 ± 10.68 ^ab^	67.78 ± 5.58 ^b^
AP	mg/kg	19.56 ± 0.90 ^a^	20.50 ± 0.66 ^a^	15.38 ± 3.35 ^a^
AK	mg/kg	70.02 ± 23.39 ^a^	125.31 ± 21.63 ^a^	78.36 ± 19.60 ^a^

Soil properties from the three time-series sugarcane fields and values followed by different small letters in the same row show significant differences (LSD test, *p* < 0.05). TN, total nitrogen; TK, total potassium; TP, total phosphorus; TS, total sulfur; OM, Organic matter; AN, available nitrogen; AK, available potassium; AP, available phosphorus; C/N, C:N ratio; NCC: first-year monoculture; CC10: ten-year continuous monoculture; CC30: thirty-year continuous monoculture.

**Table 3:** ANOSIM analysis between sugarcane cropping times.

	Bacteria		Fungi		AMF	
	*R*	*P*	*R*	*P*	*R*	*P*
NCC vs. CC10	0.559	0.022 ^∗^	0.6718	0.02 ^∗^	0.4667	0.039 ^∗^
NCC vs. CC30	0.8564	0.02 ^∗^	0.5487	0.016 ^∗^	0.3436	0.048 ^∗^
CC10 vs. CC30	0.4815	0.207	0.4444	0.109	−0.1481	0.812

Analysis of similarity was calculated between all treatments based on OTUs tables. Each pair of comparisons of two sugarcane planting times was performed using 999 permutations. NCC: first-year monoculture; CC10: ten-year continuous monoculture; CC30: thirty-year continuous monoculture. *R* values > 0.05 are generally perceived as separated, *R* > 0.75 as fully separated and *R* < 0.25 as groups hardly separated. * *p* < 0.05.

## Data Availability

Bacterial 16S rDNA gene, fungal 18S rDNA gene and Arbuscular mycorrhizae gene sequencing data were uploaded to the NCBI SRA database, with accession SRR15680451~SRR15680483.

## Supplementary Materials

The following are available online at https://www.mdpi.com/article/10.3390/microorganisms9102008/s1, Figure S1: Rarefaction curves of bacterial, fungal and AM fungal communities based on observed OTUs at 97% sequence similarity. Microbial Venn diagrams of sugarcane for different cultivation years; Table S1: Sequencing reads and coverage of bacteria, fungi and AMF of each sample derived from three time-series sugarcane fields; Table S2: The Pearson’s correlation between soil properties and microbiome genera; Table S3: Pearson’s correlation coefficients among microbial taxa at phylum level and time of sugarcane monoculture; Table S4: Pearson’s correlation coefficients among microbial genus and time of sugarcane monoculture.
