# Supplementary material for: Continuous Sugarcane Planting Negatively Impacts Soil Microbial Community Structure, Soil Fertility, and Sugarcane Agronomic Parameters
Source: Microorganisms. 2021 Sep 23;9(10):2008. doi: 10.3390/microorganisms9102008 (PMC8537732; doi:10.3390/microorganisms9102008)
Supplement: Supplementary file 1 [file microorganisms-09-02008-s001.zip › microorganisms-1393975-supplementary.pdf]

Supplementary material

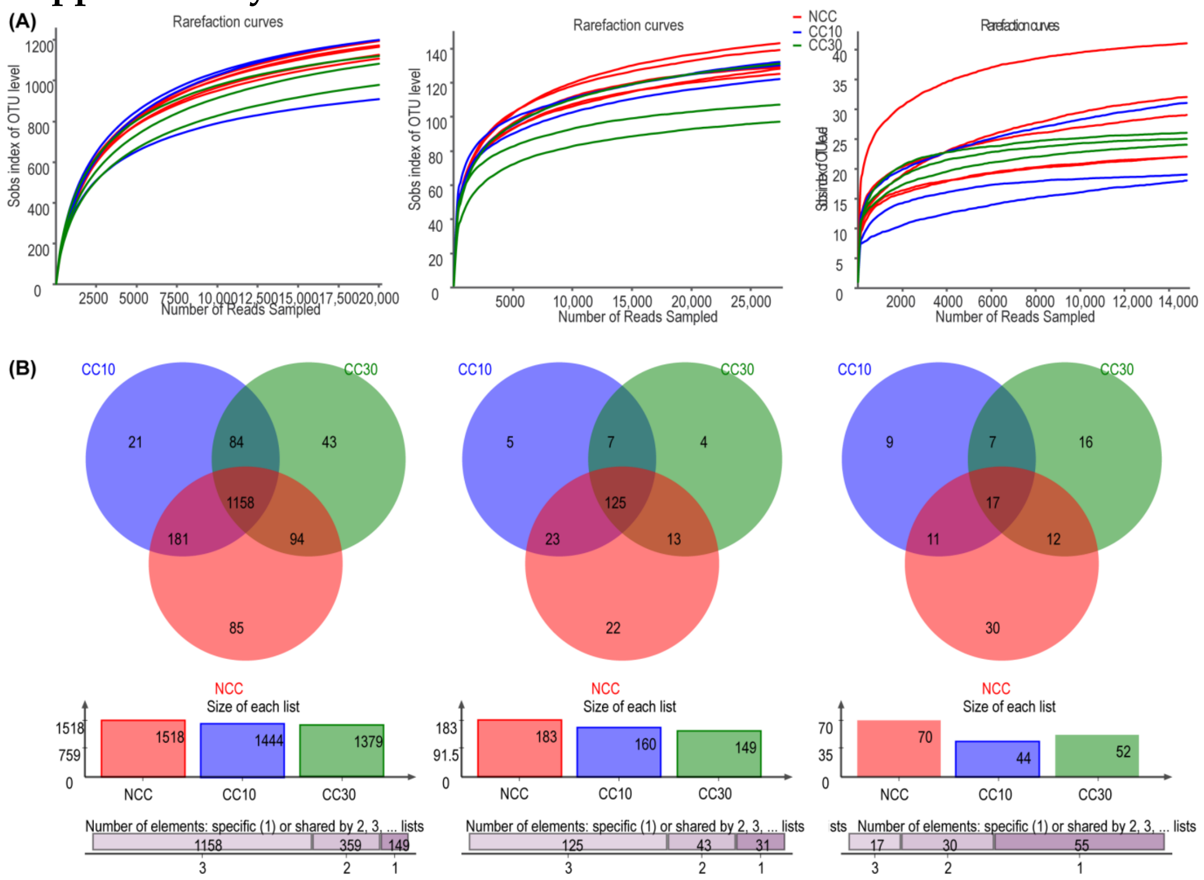

Figure S1. Rarefaction curves of bacterial, fungal and AM fungal communities based on observed OTUs at 97% sequence similarity. Microbial Venn diagrams of sugarcane for different cultivation years.

Table S1. Sequencing reads and coverage of bacteria, fungi and AMF of each sample derived from three time-series sugarcane fields.

	Sample\Info	Seq num	Base num	Mean length	Min length	Max length	OTU numbers
Bacterial	NCC_1	40459	17693743	437.3252676	313	493	1199
	NCC_2	39869	17368868	435.6484487	304	486	1199
	NCC_3	39944	17450525	436.8747497	295	483	908
	NCC_4	48262	21084591	436.8776885	281	498	1120
	NCC_5	45930	20042978	436.380971	366	488	1082
	CC10_1	37342	16240875	434.9224734	338	502	978
	CC10_2	40162	17472672	435.0548279	279	525	1126
	CC10_3	45236	19751945	436.6421655	267	507	1165
	CC30_1	35950	15628968	434.7418081	348	498	1194
	CC30_2	39159	17034825	435.0168544	267	479	1107
	CC30_3	50036	21747677	434.6405988	348	488	1172
	SUM	462349	201517667	435.829623	310	495	1666
Fungal	NCC_1	44240	17753574	401.3014014	254	433	130
	NCC_2	41199	16544004	401.5632418	277	436	132

	NCC_3	42961	17239718	401.287633	277	426	122
	NCC_4	46382	18618110	401.4080893	350	445	131
	NCC_5	49396	19825079	401.3498866	278	453	97
	CC10_1	36104	14493932	401.4494793	347	441	107
	CC10_2	43745	17576541	401.795428	236	434	129
	CC10_3	42099	16922624	401.9721134	255	435	125
	CC30_1	47077	18923746	401.9743399	329	434	128
	CC30_2	44227	17752214	401.3886088	388	422	139
	CC30_3	85372	34319599	402.0006442	378	437	143
	SUM	522802	209969141	401.5900787	306	436	199
AMF	NCC_1	24325	5814318	239.0264337	232	259	29
	NCC_2	23886	5731279	239.943021	215	259	32
	NCC_3	21977	5276204	240.0784456	233	294	41
	NCC_4	15022	3588441	238.8790441	228	258	22
	NCC_5	23787	5698639	239.5694707	224	285	22
	CC10_1	18400	4401945	239.2361413	225	272	18
	CC10_2	18184	4358251	239.675044	234	272	31
	CC10_3	24263	5768695	237.7568726	229	258	19
	CC30_1	20922	5003541	239.1521365	215	256	25
	CC30_2	23189	5552401	239.4411574	228	272	24
	CC30_3	24891	5927043	238.1199229	235	274	26
	SUM	238846	57120757	239.1706991	227	269	102

Table S2. The Pearson's correlation between soil properties and microbiome genera

soil properties	genus	R value	p value	Belonging
AK	*Fusarium*	-0.79	0.014	fungi
AN	*Ambrosiella*	0.83	0.014	fungi
AN	*Chaetomium*	0.82	0.014	fungi
AN	*Coniochaeta*	0.74	0.022	fungi
AN	*Gaiella*	0.77	0.023	bacterial
AN	*Jatrophihabitans*	-0.72	0.023	bacterial
AN	*Mrakia*	0.8	0.014	fungi
AN	norank_f__Methyloligellaceae	0.75	0.023	bacterial
AN	norank_f__Xanthobacteraceae	0.72	0.023	bacterial
AN	*Pleurothecium*	0.72	0.026	fungi
AN	unclassified_f__Aspergillaceae	-0.78	0.014	fungi
AN	unclassified_f__Micrococcaceae	0.71	0.024	bacterial
AP	Allorhizobium-Neorhizobium-Pararhizobium-Rhizobium	-0.71	0.024	bacterial

AP	norank_f__JG30-KF-AS9	0.7	0.025	bacterial
AP	unclassified_f__Sporidiobolaceae	0.79	0.014	fungi
C/N	*Coniochaeta*	0.79	0.014	fungi
C/N	*Pleurothecium*	0.78	0.014	fungi
C/N	unclassified_o__Eurotiales	0.79	0.014	fungi
OM	*Chaetomium*	0.75	0.02	fungi
OM	*Coniochaeta*	0.85	0.014	fungi
OM	*Gaiella*	0.8	0.023	bacterial
OM	norank_f__Methyloligellaceae	0.75	0.023	bacterial
OM	norank_f__SC-I-84	0.75	0.023	bacterial
OM	norank_f__Xanthobacteraceae	0.7	0.025	bacterial
OM	*Pleurothecium*	0.83	0.014	fungi
pH	*Arthrinium*	-0.7	0.029	fungi
pH	*Aureobasidium*	0.78	0.014	fungi
pH	*Coniochaeta*	0.82	0.014	fungi
pH	*Gaiella*	0.75	0.023	bacterial
pH	norank_o__Chloroplast	-0.7	0.025	bacterial
pH	*Pleurothecium*	0.84	0.014	fungi
pH	unclassified_f__Aspergillaceae	-0.71	0.027	fungi
Time	*Coniochaeta*	-0.77	0.015	fungi
Time	*Diversispora*	-0.89	0.002	AMfungi
Time	*Gaiella*	-0.88	0.009	bacterial
Time	*Jatrophihabitans*	0.76	0.023	bacterial
Time	norank_c__KD4-96	-0.72	0.023	bacterial
Time	norank_c__Sordariomycetes	-0.71	0.027	fungi
Time	norank_c__Subgroup_6	-0.73	0.023	bacterial
Time	norank_f__67-14	-0.73	0.023	bacterial
Time	norank_f__Coleofasciculaceae	0.76	0.023	bacterial
Time	norank_f__Methyloligellaceae	-0.83	0.017	bacterial
Time	norank_f__SC-I-84	-0.85	0.012	bacterial
Time	norank_f__Stigmatodiscaceae	0.77	0.015	fungi
Time	norank_f__Xanthobacteraceae	-0.82	0.018	bacterial
Time	norank_o__Chloroplast	0.87	0.009	bacterial
Time	norank_o__IMCC26256	-0.73	0.023	bacterial
Time	*Pleurothecium*	-0.86	0.014	fungi
Time	*Pseudomonas*	-0.78	0.023	bacterial
Time	*Sistotrema*	0.79	0.014	fungi
Time	unclassified_o__Agaricales	0.83	0.014	fungi
TK	*Acidothermus*	-0.91	0.007	bacterial
TK	*Dyella*	-0.74	0.023	bacterial
TK	*Kitasatospora*	-0.7	0.025	bacterial
TK	*Mucilaginibacter*	-0.7	0.025	bacterial
TK	*Pinnularia*	0.7	0.03	fungi
TN	*Acaulospora*	0.82	0.007	AMfungi
TN	*Nocardioides*	0.86	0.009	bacterial
TP	*Kitasatospora*	0.72	0.023	bacterial
TP	norank_o__Pleosporales	-0.79	0.014	fungi
TS	*Catenulispora*	-0.73	0.023	bacterial
TS	*Diversispora*	0.81	0.007	AMfungi
TS	*Flavobacterium*	0.72	0.023	bacterial
TS	*Gaiella*	0.73	0.023	bacterial

TS	norank_c__KD4-96	0.74	0.023	bacterial
TS	norank_c__Sordariomycetes	0.72	0.027	fungi
TS	norank_f__Colpodea	0.76	0.016	fungi
TS	norank_f__Methyloligellaceae	0.75	0.023	bacterial
TS	norank_f__SC-I-84	0.77	0.023	bacterial
TS	norank_f__Stigmatodiscaceae	-0.79	0.014	fungi
TS	norank_f__Xanthobacteraceae	0.74	0.023	bacterial
TS	*Sistotrema*	-0.7	0.029	fungi
Yield	*Diversispora*	0.75	0.016	AMfungi
Yield	*Flavobacterium*	0.72	0.023	bacterial
Yield	norank_f__Methyloligellaceae	0.73	0.023	bacterial
Yield	norank_f__Stigmatodiscaceae	-0.78	0.014	fungi
Yield	norank_f__Xanthobacteraceae	0.73	0.023	bacterial
Yield	*Pleurothecium*	0.76	0.016	fungi
Yield	*Sistotrema*	-0.73	0.024	fungi
Yield	unclassified_c__Glomeromycetes	-0.71	0.024	AMfungi
Yield	unclassified_o__Eurotiales	0.77	0.015	fungi

Table S3. Pearson's correlation coefficients among microbial taxa at phylum level and time of sugarcane monoculture

Bacterial and Fungal phylum	Time	Average RA (%)
Proteobacteria	-0.12	37.10
Actinobacteria	-0.38	31.66
Acidobacteria	-0.12	8.46
Chloroflexi	-0.05	5.11
Bacteroidetes	0.31	5.00
Firmicutes	-0.36	4.36
Saccharibacteria	0.57	2.00
Cyanobacteria	0.64*	1.91
Gemmatimonadetes	-0.41	1.79
Verrucomicrobia	-0.14	0.81
Ascomycota	0.23	70.89
Basidiomycota	-0.17	20.38

Pearson's correlation coefficients between the time of sugarcane monoculture ("NCC", "CC10", "CC30") and microbial taxa at the phylum level. "NCC", "CC10"and "CC30" represent three sugarcane fields with 1, 10 and 30 years of continuous cropping history, respectively.

Table S4. Pearson's correlation coefficients among microbial genus and time of sugarcane monoculture.

Bacterial abundant genera	Time	Average (%)	Fungal abundant genera	Time	Average (%)
Bradyrhizobium	0.19	3.31	*Fusarium*	-0.12	15.2
Burkholderia-Paraburkholderia	0.04	3.23	*Talaromyces*	-0.18	7.76
Bacillus	-0.42*	3.02	*Cladosporium*	-0.02	6.45
Sphingomonas	0.62*	2.67	*Coniochaeta*	-0.56*	3.80
Streptomyces	-0.17	2.6			

Pearson's correlation coefficients between the time of sugarcane monoculture ((“NCC”, “CC10”, “CC30”) and microbial genera. “NCC”, “CC10”and “CC30′′ represent three sugarcane fields with 1, 10 and 30 years of continuous cropping history, respectively.
